# Survival of neonates in rural Southern Tanzania: does place of delivery or continuum of care matter?

**DOI:** 10.1186/1471-2393-12-18

**Published:** 2012-03-23

**Authors:** Rose Nathan, Mathew Alexander Mwanyangala

**Affiliations:** 1 Ifakara Health Institute, Box 78373, Kiko Avenue, Dar es salaam, Ifakara, Tanzania

## Abstract

**Background:**

The concept of *continuum of care *has recently been highlighted as a core principle of maternal, newborn and child health initiatives, and as a means to save lives. However, evidence has consistently revealed that access to care during and post delivery (intra and postpartum) remains a challenge in the *continuum of care *framework. In places where skilled delivery assistance is exclusively available in health facilities, access to health facilities is critical to the survival of the mother and her newborn. However, little is known about the association of place of delivery and survival of neonates. This paper uses longitudinal data generated in a Health and Demographic Surveillance System in rural Southern Tanzania to assess associations of neonatal mortality and place of delivery.

**Methods:**

Three cohorts of singleton births (born 2005, 2006 and 2007) were each followed up from birth to 28 days. Place of birth was classified as either "health facility" or "community". Neonatal mortality rates were produced for each year and by place of birth. Poisson regression was used to estimate crude relative risks of neonatal death by place of birth. Adjusted ratios were derived by controlling for maternal age, birth order, maternal schooling, sex of the child and wealth status of the maternal household.

**Results:**

Neonatal mortality for health facility singleton deliveries in 2005 was 32.3 per 1000 live births while for those born in the community it was 29.7 per 1000 live births. In 2006, neonatal mortality rates were 28.9 and 26.9 per 1,000 live births for deliveries in health facilities and in the community respectively. In 2007 neonatal mortality rates were 33.2 and 27.0 per 1,000 live births for those born in health facilities and in the community respectively. Neonates born in a health facility had similar chances of dying as those born in the community in all the three years of study. Adjusted relative risks (ARR) for neonatal death born in a health facility in 2005, 2006 and 2007 were 0.99 (95%CI: 0.58 - 1.70), 0.98 (95%CI: 0.62 - 1.54) and 1.18 (95% CI: 0.76 - 1.85) respectively.

**Conclusions:**

We found no evidence to suggest that delivery in health facilities was associated with better survival chances of the neonates.

## Background

In recent years health of the neonates has increasingly received attention as a critical component in child survival. Publications in the Lancet series that focused on neonatal health and the 2005 World Health report provided unprecedented evidence of the magnitude and importance of neonatal mortality particularly in the developing countries [1-4].

Among other things, the papers brought to light the fact that a large proportion of child deaths occur during neonatal period and as a result of interventions targeting children after neonatal period, proportion of deaths that occur in the neonatal period has increased [1]. Despite the gains in under-five mortality over the past years, it has become evident that MDG 4 that targets reduction of under-five mortality by two thirds by 2015 is unlikely to be achieved if neonatal survival chances do not improve [1]. Recent evidence has shown a comparatively little drop in neonatal mortality rates in Africa between 1990 and 2009 [5]. In Africa, the drop was only 17.6% (from 43.6 to 35.9 per 1,000 live births), while in some regions the rates halved over that period.

In Tanzania, the 2004-5 DHS estimates showed a statistically significant decline of under-five mortality from 147 per 1,000 live births in the late 90s to 112 per 1,000 live births in 2000-2004, likewise infant mortality dropped from 99 to 68 over the same time period but neonatal mortality remained above 30 per 1000 live births [6]. The gains in child survival are mainly attributed to effective interventions such as IMCI that target post neonatal age [7].

Due to the intrinsic link between the health of the mother and her newborn as well as realisation that the overall lifecycle ultimately determine the health of a pregnant woman and her newborn, continuum of care is fundamentally considered the most effective strategy in improving both maternal and neonatal health[4]. Within the *continuum of care *framework, a concern shared across many low-income countries is the low coverage of interventions during delivery and postnatal care despite high utilisation of antenatal care [8]. In Tanzania, according to the latest Demographic & Health Survey report, over 90 percent of pregnant women received antenatal care from a health professional at least once, but only 50 per cent gave birth in health facilities[9]. This raises concern particularly in Tanzania where skilled attendance is synonymous with facility delivery. Studies that explored barriers to obstetric care in various parts of Tanzania singled out poor quality of care as one of the important limiting factors [10,11].

Assessment of the neonatal survival benefit conferred by institutional delivery over unsupervised community delivery can potentially inform the health delivery system. In countries where the coverage of institutional delivery is high, neonatal mortality is comparatively lower [1]. However, studies that provide evidence of a direct link between institutional delivery and neonatal survival are scarce particularly at sub-national level. The National Demographic and Health surveys, the main source of child mortality are limited in their sample sizes - too small to produce precise estimates at lower levels such as a district, a unit where in several countries including Tanzania policies are translated into action.

Health and Demographic Surveillance Systems that are operational in several African countries can be a potential source for assessing neonatal survival gains acquired through delivering in a health facility at district level. This paper uses longitudinal data generated in a Health and Demographic Surveillance System in rural Southern Tanzania to assess associations of neonatal mortality and place of delivery.

## Methods

This study was done in Ifakara Health and Demographic Surveillance Site located in Southern Tanzania, Morogoro region. The HDSS site was started by conducting baseline census between September and December 1996. Since then every household in the surveillance area has been visited by a trained interviewer every 4 months to record pregnancies, pregnancy outcomes, deaths and migrations that have happened since the previous visit. Date of birth of each individual is included in the household registers and each event is recorded along with specific date it happened. Place of delivery and place of death are recorded as health facility, home or elsewhere. Educational levels of each individual and household assets are recorded annually. Currently (2011), the site includes over 100,000 people living in 25 villages in parts of two districts, Kilombero and Ulanga in Southern Tanzania. The population is predominantly rural and ethnically heterogeneous. Majority of the households earn their living from subsistence farming, few are engaged in fishing and small-scale trading. Detailed description of the study area is presented elsewhere [12].

The population of the study districts is served by a network of health facilities, at the time of the study there were two hospitals, four health centres and twenty one dispensaries in Kilombero district; two hospitals, three health centres and twenty dispensaries in Ulanga district. In 2008, comprehensive EMOC was available in two hospitals in each district. Health facilities with staff available for 24 hours, 7 days per week to perform normal delivery were only 59% and 72% in Kilombero and Ulanga districts, respectively. Within the study population, about 60% of all deliveries occur in health facilities mainly in dispensaries. Use of antenatal services by women in the study area is over 95% (at least one visit to ANC clinic). At the time of study, continuum of care was not fully introduced in the study area.

This paper reports analysis of observational data collected in the Ifakara Health and Demographic Surveillance Site (IHDSS) for children born between 2005 and 2007. Three birth cohorts of singleton neonates were extracted from the database including their survival status within the first 28 days of life. Variables of interest included date of birth, date of death, birth order, sex, maternal age at birth, maternal education, household economic status, place of delivery and place of death.

Data credibility was ensured at all stages of collection and processing. Up to 5% of randomly selected households were visited by field supervisors for repeated interviews. Other strategies included accompanied interviews as well as surprise field visits by field managers. Data was keyed in computers using a household registration system (HRS), software for relational database with inbuilt consistency and range checks. Captured inconsistencies were referred back to the field.

Neonatal death is defined as termination of life of a live-born child within 28 days of life. Place of delivery is classified as "in the health facility" or "in the community". Health facility includes dispensaries, health centres and hospitals. Delivery at home, TBAs homes or anywhere else besides health facilities are classified here as "in the community". We included in this paper only singleton live births that occurred between year 2005 and 2007.

Neonatal mortality was calculated as the number of neonatal deaths divided by number of live births in a given year and expressed per 1000 live births. Mortality on the same day of life was calculated as the number of neonates that had date of birth same as date of death divided by number of live births in a given year and expressed per 1000 live births.

We calculated means and percentages of the background characteristics and performed *t *tests for means and χ^2 ^tests for proportions to asses differences in the maternal and child background characteristics between the two defined places of delivery (health facility, community).

For each year of study, Poisson regression models were fitted to estimate crude relative risks of neonatal death by place of delivery. Adjusted ratios were derived by controlling for maternal age, birth order, maternal schooling, sex of the child and wealth status of the maternal household.

Daily survival functions of the neonates born in health facilities and those born in the community were estimated and compared using log rank tests.

Ifakara Health and Demographic Surveillance System was established with an initial aim of evaluating the effect of a large-scale social marketing of insecticide-treated nets on child survival in rural Tanzania. The study was approved by local and national ethical committees.

## Results

There were a total of 2852, 2851 and 2890 singleton live births in 2005, 2006 and 2007 respectively. Of those, 58.6%, 58.9% and 61.5% occurred in health facilities. Most of the births occurred to women aged between 20 and 30 years, teenagers contributed about 20% of all the births. Over 10% of the births were in the order of 6 or above. Most of the women (> 55%) had completed primary education and only about 2% had attained education beyond primary level. Percentage distribution of births by socio-economic status indicated that deliveries in health facilities were more clustered in the higher quintiles of socio-economic status (Table 1).

**Table 1:** Maternal background characteristics

Background characteristics	2005	2006	2007
**Age**			
< 20	503 (17.6%)	526 (18.5%)	489 (16.9%)
20-34	1912 (67.1%)	1908 (66.9%)	2058 (71.2%)
> 34	437 (15.3%)	417 (14.6%)	343 (11.9%)
**Parity**			
Prim	525 (18.4%)	563 (19.8%)	553 (19.2%)
2-6	1994 (69.9%)	1974 (69.2%)	2047 (70.8%)
> 6	333 (11.7%)	314 (11.0%)	290 (10.0%)
**Education**			
None	317 (15.8%)	458 (16.1%)	467 (16.1%)
Primary incomplete	509 (25.4%)	781 (26.7%)	678 (23.5%)
Primary complete	1151 (57.3%)	1577 (55.3%)	1687 (58.4%)
Beyond Primary	31 (1.5%)	55 (1.9%)	58 (2.0%)
**Socio-economic status**			
Poorest	539 (18.9%)	534 (15.2%)	463 (16.0%)
Q2	588 (20.6%)	538 (18.9%)	543 (18.8%)
Q3	502 (17.6%)	777 (27.3%)	678 (23.5%)
Q4	606 (21.3%)	598 (20.9%)	572 (19.8%)
Least poor	617 (21.6%)	504 (17.7%)	634 (21.9%)
**Total**	**2852**	**2851**	**2890**

Analysis of maternal and child characteristics by place of delivery showed that women who delivered in the community were consistently slightly older (p = < 0.01) and had a higher parity in each of the three years of the study (p = < 0.01). Incidentally higher proportions of male neonates were born in health facilities but the difference was not statistically significant. Women who delivered in health facilities had a slightly higher mean number of years of schooling but the difference was less than one year in each of the three years (Table 2).

**Table 2:** Place of delivery by background characteristics

			Year			
	
Background characteristics	2005		2006		2007	
	
	Health facility	Community	Health facility	Community	Health facility	Community
**Maternal and Newborn**						
Maternal age (mean, (95% CI)	26.5 (26.2-26.8)	27.5 (27.1-27.9)	26.6 (26.3-26.9)	27.7 (27.3-28.1)	26.6(26.2-26.9)	27.7 (27.3-28.1)
Birth order (mean, 95% CI)	3.5(3.3-3.6)	3.8 (3.7-3.9)	3.3 (3.2-3.4)	3.8 (3.7-3.9)	3.3 (3.2-3.5)	3.9 (3.7-4.0)
Education (mean years, 95% CI)	5.4 (5.2-5.5)	4.9 (4.7-5.1)	5.4 (5.2-5.5)	5.1 (4.9-5.3)	5.4 (5.2-5.5)	4.9 (4.7-5.1)
Male child	52.3%	47.5%	51.9%	49.7%	52.2%	51.6%
**Household socio-economic**						
**status**						
Poorest	17%	21%	14%	18%	14%	19%
Q2	19%	23%	18%	19%	19%	19%
Q3	18%	16%	26%	29%	23%	24%
Q4	22%	21%	21%	21%	20%	19%
Least poor	24%	18%	20%	13%	24%	19%
**Total number of live births** **(singleton)**	**1672**	**1180**	**1697**	**1154**	**1777**	**1113**

A separate analysis showed that twins were just as likely to be born in the community as were singletons. A total of 323 twins were recorded in the 3 years of the study, of them 124 (38%) were born in the community. This compared closely with 40% of singleton births delivered in the community.

First- day of life death rates were higher for neonates born in the community for 2005 and 2006 deliveries but in 2007 those born in health facilities died at a much higher rate compared to those born in the community (15 per thousand live births in the health facility and 9 per thousand live births in the community). Overall, neonates born between 2005 and 2007 faced a very high risk of dying during the first day of life independent of place of birth. The neonates in each of the two groups (born in health facility and those born in the community) experienced a first-day of life mortality of 13 per thousand live births (Table 3).

**Table 3:** Neonatal death within the same day of life

				Year				
	
Births and neonatal deaths		2005		2006		2007		Total
	
	HF*	Community	HF	Community	HF	Community	HF	Community
Total live births	1672	1180	1697	1154	1777	1113	5146	3447
Total neonatal deaths	64	40	64	38	60	33	188	111
Died the same day of delivery	22	20	16	15	27	10	65	45
Mortality rate within the same	13	17	9	13	15	9	13	13
day of life (per 1000 live births)								

* Health facility

Overall neonatal mortality rates for singletons were 31.2, 28.1 and 30.8 per 1,000 live births in 2005, 2006 and 2007 respectively. A slight decline was observed between 2005 and 2006 (9%) but rebounded in 2007. While there was a small decline (9.1%) of neonatal mortality for births that occurred in the community between 2005 and 2007, a slight increase (2.9%) was observed for neonates born in the health facilities (Table 4).

**Table 4:** Relative risk of neonatal death among singleton

Year	Health Facility		Community		Relative risk (facility against community)		p value
		
	Live births	NMR	Live births	NMR	Crude (95% CI)	Adjusted (95% CI)	
2005	1672	32.3	1180	29.7	1.09 (0.71-1.66)	0.99 (0.58-1.70)	0.99
2006	1697	28.9	1154	26.9	1.07 (0.69-1.69)	0.98 (0.62-1.54)	0.92
2007	1777	33.2	1113	27.0	1.23 (0.79-1.91)	1.18 (0.76-1.85)	0.46

Estimates of crude relative risks showed that children born in health facilities were at a similar risk of a neonatal death to those born in the community (Table 4). Adjusted relative risks were: 0.99(95% CI 0.58-1.70), 0.98 (95% CI: 0.62-1.54) and 1.18 (95% CI: 0.76-1.85) for 2005, 2006 and 2007 respectively.

**Figure 1:**
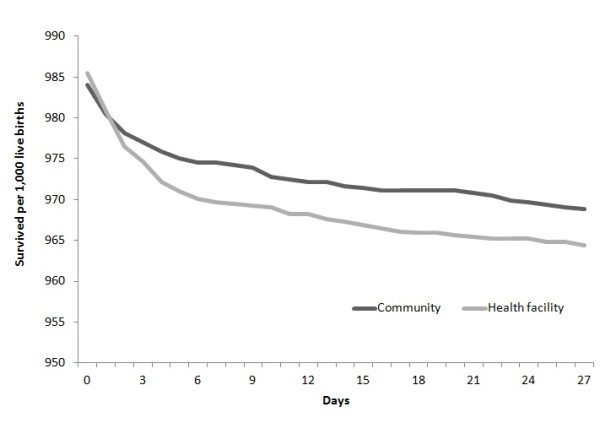
Daily survival of neonates by place of birth

Assessment of daily survival by place of delivery (Figure 1) showed that, children born in the community had similar survival rates to those born in health facilities (χ2 = 1.39, p = 0.238).

Tabulation of place of death by place of delivery indicated that one third of the neonatal deaths among those delivered in health facilities happened after discharge and 29% of those born in the community died in a health facility.

## Discussion

We used a Health and Demographic Surveillance data to estimate place of birth-specific neonatal mortality rates for three consecutive years to explore whether there was evidence of survival benefits gained by delivering in a health facility. This study is done in a rural setting where ANC, delivery care and postnatal services are provided within the formal health delivery system as per the Ministry of Health and Social Welfare guidelines - focused Antenatal Care Package based on the World Health Organisations (WHO), the model was introduced in 2002 [13]. In the study area, EMOC services have low coverage but use of ANC services is very high. Over 95% of pregnant women make at least one visit to antenatal care clinic. In the study population, almost all deliveries that happen in the community are not assisted by a skilled attendant. This phenomenon is not unique to the study area, Ronsmans *et al *[14] documented similar observation in West Africa where nearly all births with skilled attendant took place in a health facility. At the time of the study, coverage of deliveries in health facilities in the study area was higher than the current national average (60% Vs 50%).

Analysis of the Ifakara HDSS delivery data for three consecutive years revealed that, on average women who delivered in health facilities were younger, had lower parity, had a higher mean number of years of education and were more likely to live in households of higher socio-economic status. Most of these attributes are favourable for survival of a newborn, however, throughout the study period, neonates delivered in health facilities experienced a similar level of mortality to those born in the community. Results were observed consistently across three indicators used to assess mortality; overall mortality, life table survival functions and relative risks of a neonatal death.

We found no evidence to suggest that delivery in health facilities is protective to the newborns. These results are contrary to the expected neonatal survival gains conferred through institutional delivery [1]. Lack of information about factors such as complications in pregnancy and gestational age that might have influenced choice of place of delivery can be a potential source of bias. Birth weight as an important determinant of neonatal survival is also missing in this study. However, systematic selection of place of delivery was not evident as we noted that multiple deliveries (one of risk indicators) happened in the community at a proportion comparable to singleton deliveries (38% Vs 40%). A study in Indonesia that controlled for birth size and delivery complications reported a protective role of institutional deliveries in urban areas but increased risk associated with deliveries in public hospitals in rural areas [15].

Another dimension to the observed results is the poor quality of care in health facilities. A recent systematic review indicated that over three quartets of intrapartum-related deaths occurred in settings with weak health systems [16]. Scarcity of skilled providers, poor infrastructure and substandard quality of care are some of the critical components of such health systems that constrain progress in maternal and newborn survival [17]. A national survey that assessed service provision in Tanzania revealed serious challenges facing the health facilities including low coverage of the most basic infection-control items such as washing soap (59%), running water (38%) and latex gloves (50%) [18]. Considering that infections including sepsis contribute substantially to the causes of neonatal mortality [19,20], unhygienic conditions in the health facilities are potentially responsible for part of the neonatal deaths.

Shortage of skilled providers in Tanzania remains critical. In 2004, the government declared a crisis in Human Resources for Health [21]. Analysis of workforce in the 68 countdown priority countries using the density of physicians, nurses and midwives indicators showed that Tanzania ranked tenth from bottom in coverage of such cadre [22].

A recent study in another region in Southern Tanzania explored perception and perspectives of women and care providers about use of antenatal and postnatal care. The study documented complaints from the women about shortage of basic equipment, supplies and staff [23]. Such situation is reflective in the observed high mortality rate within the first day of life. Lack of adequate maternal and neonatal care at that critical time has been argued to be linked to deaths within the first day of life [19]. Findings from facility-based studies in parts of north eastern Tanzania that assessed unmet need for emergency obstetric care blamed poor quality of care for the negative maternal outcomes and high perinatal mortality [24,25].

Strengthening health systems in Tanzania is critical in saving the lives of newborns but should happen in an integrated approach as stipulated in the concept of *continuum of care *that has recently been highlighted as a core principle of maternal, newborn and child health programme, and as a means to save their lives [4,26]. *Continuum of car*e identifies three key delivery approaches: facility-based clinical care, outreach and the third one is family and community care that consists of home-based care and practices. It is apparent that each of the three approaches is necessary but none is sufficient on its own but synergistic connections are crucial for making an impact. Our findings support the emphasized need for linkages between community and facility-based care in the context of *continuum of care*. The one third of the neonates that died at home after delivery in the health facility communicates an important message that "delivery in a health facility alone without effective care in the antenatal period is not enough to prevent death of a newborn". Appropriate home management of the newborn after delivery in the health facility and access to facility-based clinical care in case a need arises are critical to the survival of the newborn. It has been established that 90% coverage of facility-based clinical care alone could reduce neonatal mortality by 23-50% [2].

Maternal and newborn community-based intervention trials conducted in several Asian countries have provided evidence of efficacy of such interventions in improving neonatal survival [27-29]. Notwithstanding the contextual differences between Tanzania and the communities where those trials have been conducted, they offer useful practical examples that can be tested and adapted to suit the culture, behaviour and health policies and practices in Tanzania. Strengthening the links along the community to the health facilities in the framework of *continuum of care *is a critical component of the success of such interventions. Encouragingly, the importance of community-based maternal and newborn health interventions as a crucial compliment to the facility-based care is well stipulated in the Tanzania National Road map - a strategic plan to accelerate reduction of maternal and newborn deaths [30].

The study findings cannot be generalised due to variability of the quality of maternal and newborn health care across various sub groups within Tanzania.

## Conclusions

Findings of this study calls for investing in improving the quality of services and strengthening health system in general alongside exploring strategies to make delivery in the community safer to the mother and her newborn. This study covers the period just prior to the implementation of the strategic plan to accelerate reduction of maternal and newborn deaths in Tanzania. As such it could be considered as setting a baseline for the study area over which the impact of the initiatives on neonatal survival could be monitored and evaluated as more and more deliveries are expected to happen in quality-improved health facilities.

## Competing interests

The authors declare that they have no competing interests.

## Authors' contributions

RN performed all data analysis and interpretation of the results, drafting and revising the manuscript. RN provided the final decision for submission. MAM participated in data extraction, data analysis as well as drafting and reviewing the manuscript. All authors read and approved the final manuscript.

## Pre-publication history

The pre-publication history for this paper can be accessed here:

http://www.biomedcentral.com/1471-2393/12/18/prepub
